# Supplementary material for: Prior vaccination with rVSV-ZEBOV does not interfere with but improves efficacy of postexposure antibody treatment
Source: Nat Commun. 2020 Jul 27;11:3736. doi: 10.1038/s41467-020-17446-4 (PMC7385100; doi:10.1038/s41467-020-17446-4)
Supplement: Supplementary file 1 — Supplementary Information [file 41467_2020_17446_MOESM1_ESM.pdf]

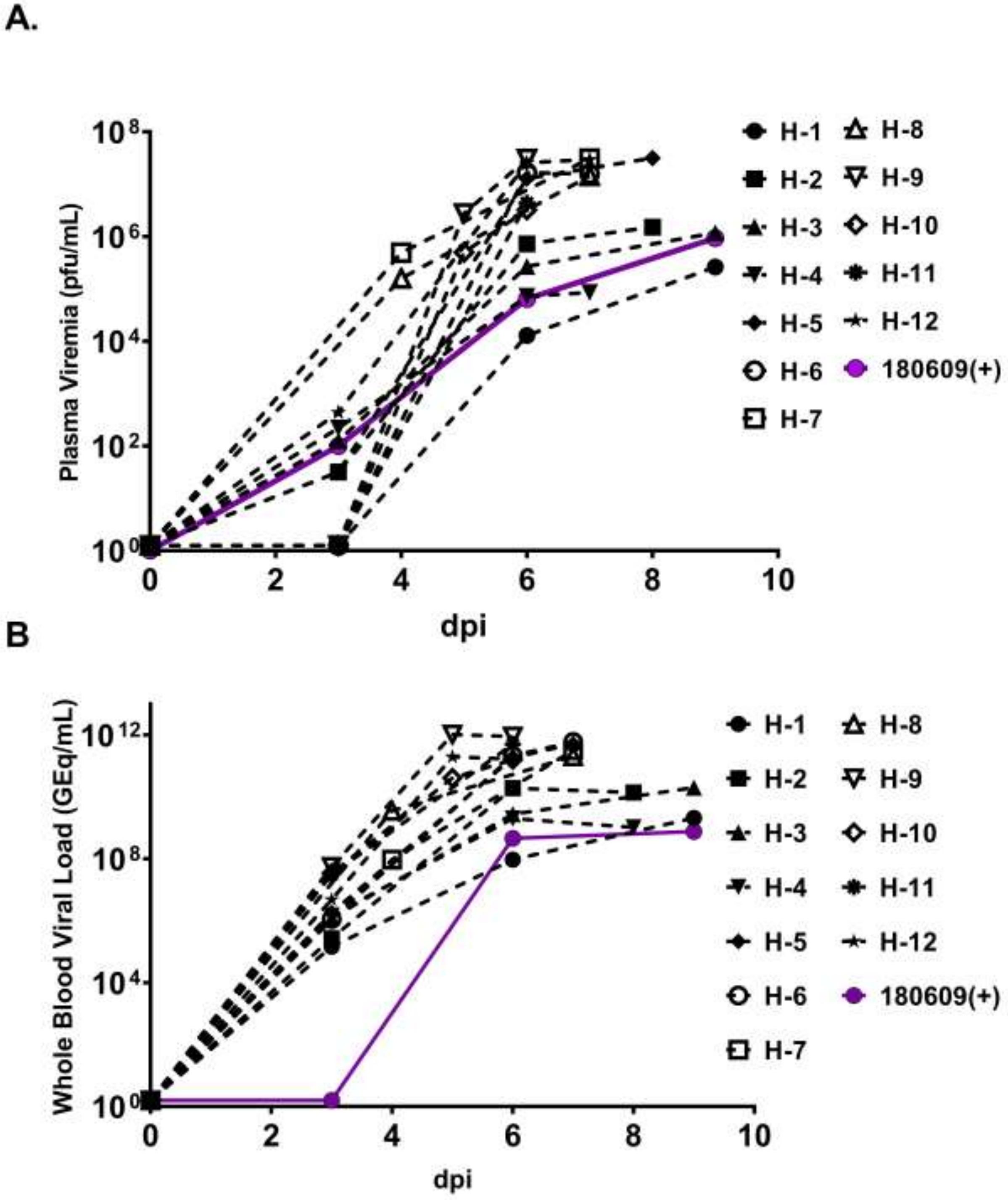

Supplementary Figure 1: Historical control rhesus macaques infected with EBOV-Kikwit: A. Infectious virus in circulation. Limit of detection is 25 pfu/ml. B. vRNA content in whole blood.

Supplementary Table 1. Clinical description and outcome of rVSV-EBOV-GP vaccinated/MIL77 treated and control rhesus macaques following EBOV challenge

Subject No.	Sex	Treatment	Clinical illness	Clinical pathology
EXP-Control	F	None	Decreased appetite (d5-7); fever (d6); anorexia (d8,9); mild petechial rash (d9); recumbency (d9). Subject euthanized on d9.	Lymphopenia (d6,9); thrombocytopenia (d6,9); monocytopenia (d9); leukocytosis (d3); monocytosis (d3); granulocytosis (d3,9); hypoalbuminemia (d9); > 4-fold ↑ ALT (d9); > 24-fold ↑ AST (d9); > 2-fold ↑ ALP (d9); > 3-fold ↑ GGT (d9); > 11-fold ↑ CRE (d9); > 17-fold ↑ CRP (d6,9)
VSV/mAb-1	F	rVSV-EBOV-GP + MIL77	Fever (d1). Subject survived to study endpoint.	Granulocytosis (d0,1,14,21,28); 2-fold ↑ CRE (d21,28)
VSV/mAb-2	F	rVSV-EBOV-GP + MIL77	None. Subject survived to study endpoint.	Lymphopenia (d28); thrombocytopenia (d6); leukocytosis (d6); monocytosis (d0,3,6,14); granulocytosis (d6); hypoglycemia (d9,28); hypoalbuminemia (d9); > 2-fold ↑ in CRP (d6,9)
VSV/mAb-3	M	rVSV-EBOV-GP + MIL77	None. Subject survived to study endpoint.	Lymphopenia (d6); monocytosis (d0,28); granulocytosis (d28); > 2-fold ↑ in ALT (d0); > 2-fold ↑ in AST (d0,1)
VSV/mAb-4	M	rVSV-EBOV-GP + MIL77	None. Subject survived to study endpoint.	Erythrocytopenia (d3,6) ; thrombocytopenia (d3); lymphopenia (d0,3,9,14,21,28); monocytopenia (d0); hypoglycemia (d6,9,28); > 2-fold ↑ in CRP (d0,6,9)
VSV/mAb-5	M	rVSV-EBOV-GP + MIL77	None. Subject survived to study endpoint.	Lymphopenia (d28); monocytosis (d3,6,9); granulocytosis (d0,1,3,6,9,28); hypoglycemia (d3,9,28); 2-fold ↑ in CRE (d3)

Days after EBOV challenge are in parentheses. Lymphopenia, granulopenia, monocytopenia, and thrombocytopenia are defined by a ≥35% drop in numbers of lymphocytes, granulocytes, monocytes, and platelets, respectively. Leukocytosis, monocytosis, and granulocytosis are defined by a two-fold or greater increase in numbers of white blood cells over base line. Fever is defined as a temperature more than 2.5 °F over baseline, or at least 1.5 °F over baseline and ≥ 103.5 °F. Hypothermia is defined as a temperature ≤3.5°F below baseline. Hyperglycemia is defined as a two-fold or greater increase in levels of glucose. Hypoglycemia is defined by a ≥25% decrease in levels of glucose. Hypoalbuminemia is defined by a ≥25% decrease in levels of albumin. Hypoproteinemia is defined by a ≥25% decrease in levels of total protein. Hypoamylasemia is defined by a ≥25% decrease in levels of serum amylase. Hypocalcemia is defined by a ≥25% decrease in levels of serum calcium. (ALT) alanine aminotransferase, (AST) aspartate aminotransferase, (ALP) alkaline phosphatase, (CRE) Creatinine, (CRP) C-reactive protein, (Hct) hematocrit, (Hgb) hemoglobin

Supplementary Table 2. Clinical description and outcome of rVSV-EBOV-GP vaccinated rhesus macaques following EBOV challenge

Subject No.	Sex	Treatment	Clinical illness	Clinical pathology
VSV-1	F	rVSV-EBOV-GP	Decreased appetite (d9,10). Subject survived to study endpoint.	Lymphopenia (d0,6,9); monocytopenia (d0); granulocytopenia (d0,1,3,6); thrombocytosis (d14); granulocytosis (d9); hypoalbuminemia (d9); > 2-fold ↑ AST (d1); hypoamylasemia (d9); > 23-fold ↑ CRP (d9)
VSV-2	M	rVSV-EBOV-GP	Fever (d6); anorexia (d8-12); petechial rash (d9-15) decreased appetite (d13-16). Subject survived to study endpoint.	Lymphocytopenia (d0,9); thrombocytopenia (d1,9,14); monocytopenia (d0,9); granulocytopenia (d1,3,9,14); thrombocytosis (d21); monocytosis (d1,6,14); hypoalbuminemia (d9); > 2-fold ↑ ALT (d28); > 7-fold ↑ AST (d9); > 2-fold ↑ ALP (d9,14,21); hypoamylasemia (d9); > 27-fold ↑ CRP (d9)
VSV-3	F	rVSV-EBOV-GP	Fever (d6,9); decreased appetite (d8-10); petechial rash (d9,10); hypothermia (d10); epistaxis (d10); recumbency (d10). Subject euthanized on d10.	Lymphocytopenia (d0,6); thrombocytopenia (d9); monocytopenia (d0); granulocytopenia (d1,3); monocytosis (d10); > 2-fold ↑ BUN (d9,10); > 5-fold ↑ CRE (d9,10); hypocalcemia (d10); hypoalbuminemia (d9,10); > 10-fold ↑ ALT (d9,10); > 73-fold ↑ AST (d9,10); > 3-fold ↑ ALP (d9,10); > 3-fold ↑ GGT (d9,10); hypoamylasemia (d6,9); > 27-fold ↑ CRP (d9,10)
VSV-4	M	rVSV-EBOV-GP	Fever (d6); decreased appetite (d6); anorexia (d7,8); hypothermia (d8); petechial rash (d8); bradypnea (d8); recumbency (d8). Subject euthanized on d8.	Lymphopenia (d0,6); thrombocytopenia (d8); monocytopenia (d0); leukocytosis (d6); granulocytosis (d6,8); hypoglycemia (d8); > 4-fold ↑ BUN (d8); > 10 ↑ CRE (d8); hypoalbuminemia (d8); > 25-fold ↑ ALT (d8); > 114-fold ↑ AST (d8); > 2-fold ↑ ALP (d8); > 3-fold ↑ GGT (d8); > 24-fold increase CRP (d6,8)
VSV-5	M	rVSV-EBOV-GP	Fever (d6); petechial rash (d7-9); decreased appetite (d7), anorexia (d8); recumbency (d8); hypothermia (d8). Subject euthanized on d8.	Lymphocytopenia (d0,6); thrombocytopenia (d6,8); monocytopenia (d0); granulocytopenia (d0,3,8); granulocytosis (d6); > 4-fold ↑ BUN (d8); > 8-fold ↑ CRE (d8); hypocalcemia (d8); > 8-fold ↑ ALT (d8); > 82-fold ↑ AST (d8); > 2-fold ↑ GGT (d8); > 22-fold ↑ CRP (d6,8)

Days after EBOV challenge are in parentheses. Lymphopenia, granulopenia, monocytopenia, and thrombocytopenia are defined by a ≥35% drop in numbers of lymphocytes, granulocytes, monocytes, and platelets, respectively. Leukocytosis, monocytosis, and granulocytosis are defined by a two-fold or greater increase in numbers of white blood cells over base line. Fever is defined as a temperature more than 2.5 °F over baseline, or at least 1.5 °F over baseline and ≥ 103.5 °F. Hypothermia is defined as a temperature ≤3.5°F below baseline. Hyperglycemia is defined as a two-fold or greater increase in levels of glucose. Hypoglycemia is defined by a ≥25% decrease in levels of glucose. Hypoalbuminemia is defined by a ≥25% decrease in levels of albumin. Hypoproteinemia is defined by a ≥25% decrease in levels of total protein. Hypoamylasemia is defined by a ≥25% decrease in levels of serum amylase. Hypocalcemia is defined by a ≥25% decrease in levels of serum calcium. (ALT) alanine aminotransferase, (AST) aspartate aminotransferase, (ALP) alkaline phosphatase, (CRE) Creatinine, (CRP) C-reactive protein, (Hct) hematocrit, (Hgb) hemoglobin

Supplementary Table 3. Clinical description and outcome of MIL77 treated rhesus macaques following EBOV challenge

Subject No.	Sex	Treatment	Clinical illness	Clinical pathology
mAb-1	F	MIL77	Fever (d6); decreased appetite (d6-10); peripheral edema (d9,10). Subject survived to study endpoint.	Lymphopenia (d3,6,9); monocytopenia (d9,21); monocytosis (d3); granulocytosis (d3,6); > 2-fold ↑ AST (d9); > 2-fold ↑ ALP (d9,14); > 24-fold ↑ CRP (d6,9)
mAb-2	M	MIL77	Decreased appetite (d7-11). Subject survived to study endpoint.	Lymphocytopenia (d3,6,9); monocytopenia (d9); leukocytosis (d6); monocytosis (d0,6); granulocytosis (d1,3,6,9,14); hypoglycemia (d14,28); hypoalbuminemia (d9,14); > 2-fold ↑ ALP (d9); hypoamylasemia (d6,9)
mAb-3	M	MIL77	Fever (d6,9); decreased appetite (d6-14). Subject survived to study endpoint.	Lymphocytosis (d3,9); monocytopenia (d3,9,14); granulocytopenia (d28); leukocytosis (d6); monocytosis (d0); granulocytosis (d3,6); hypoglycemia (d14); hypoamylasemia (d3,6,9); > 26-fold ↑ CRP (d6,9)
mAb-4	M	MIL77	Fever (d6); peripheral edema (d9); decreased appetite (d9-11). Subject survived to study endpoint.	Monocytopenia (d14); granulocytopenia (d21); leukocytosis (d9); monocytosis (d6); granulocytosis (d3,6,9); hypoglycemia (d14); hypoalbuminemia (d9,14); > 2-fold ↑ AST (d9); hypoamylasemia (d9); > 28-fold ↑ CRP (d9)
mAb-5	F	MIL77	Fever (d6); decreased appetite (d6); anorexia (d7-9); depression (d8,9); petechial rash (d6-9); tachypnea (d9); recumbency (d9). Subject euthanized on d9.	Lymphopenia (d6); thrombocytopenia (d6,9); monocytopenia (d9); granulocytopenia (d9); leukocytosis (d3); monocytosis (d0,1,3); granulocytosis (d0,1,3,6); hypoglycemia (d9); > 5-fold ↑ BUN (d9); > 4-fold ↑ BUN (d9); hypocalcemia (d9); hypoalbuminemia (d9); hypoproteinemia (d9); > 31-fold ↑ ALT (d9); > 188-fold ↑ AST (d9); > 2-fold ↑ GGT (d9); hypoamylasemia (d6,9); > 21-fold ↑ CRP (d6,9)

Days after EBOV challenge are in parentheses. Lymphopenia, granulopenia, monocytopenia, and thrombocytopenia are defined by a ≥35% drop in numbers of lymphocytes, granulocytes, monocytes, and platelets, respectively. Leukocytosis, monocytosis, and granulocytosis are defined by a two-fold or greater increase in numbers of white blood cells over base line. Fever is defined as a temperature more than 2.5 °F over baseline, or at least 1.5 °F over baseline and ≥ 103.5 °F. Hypothermia is defined as a temperature ≤3.5°F below baseline. Hyperglycemia is defined as a two-fold or greater increase in levels of glucose. Hypoglycemia is defined by a ≥25% decrease in levels of glucose. Hypoalbuminemia is defined by a ≥25% decrease in levels of albumin. Hypoproteinemia is defined by a ≥25% decrease in levels of total protein. Hypoamylasemia is defined by a ≥25% decrease in levels of serum amylase. Hypocalcemia is defined by a ≥25% decrease in levels of serum calcium. (ALT) alanine aminotransferase, (AST) aspartate aminotransferase, (ALP) alkaline phosphatase, (CRE) Creatinine, (CRP) C-reactive protein, (Hct) hematocrit, (Hgb) hemoglobin
