# Supplementary material for: Prior vaccination with rVSV-ZEBOV does not interfere with but improves efficacy of postexposure antibody treatment
Source: Nat Commun. 2020 Jul 27;11:3736. doi: 10.1038/s41467-020-17446-4 (PMC7385100; doi:10.1038/s41467-020-17446-4)
Supplement: Supplementary file 2 — Reporting Summary [file 41467_2020_17446_MOESM2_ESM.pdf]

nature research

Corresponding author(s): Thomas W. Geisbert

Last updated by author(s): Jun 22, 2020

Reporting Summary

Nature Research wishes to improve the reproducibility of the work that we publish. This form provides structure for consistency and transparency in reporting. For further information on Nature Research policies, see Authors & Referees and the Editorial Policy Checklist .

Statistics

For all statistical analyses, confirm that the following items are present in the figure legend, table legend, main text, or Methods section.

n/a	Confirmed	
☐	☒	The exact sample size (*n*) for each experimental group/condition, given as a discrete number and unit of measurement
☐	☒	A statement on whether measurements were taken from distinct samples or whether the same sample was measured repeatedly
☐	☒	The statistical test(s) used AND whether they are one- or two-sided<br>*Only common tests should be described solely by name; describe more complex techniques in the Methods section.*
☐	☒	A description of all covariates tested
☐	☒	A description of any assumptions or corrections, such as tests of normality and adjustment for multiple comparisons
☐	☒	A full description of the statistical parameters including central tendency (e.g. means) or other basic estimates (e.g. regression coefficient) AND variation (e.g. standard deviation) or associated estimates of uncertainty (e.g. confidence intervals)
☐	☒	For null hypothesis testing, the test statistic (e.g. *F*, *t*, *r*) with confidence intervals, effect sizes, degrees of freedom and *P* value noted<br>*Give P values as exact values whenever suitable.*
☒	☐	For Bayesian analysis, information on the choice of priors and Markov chain Monte Carlo settings
☒	☐	For hierarchical and complex designs, identification of the appropriate level for tests and full reporting of outcomes
☒	☐	Estimates of effect sizes (e.g. Cohen's *d*, Pearson's *r*), indicating how they were calculated

Our web collection on statistics for biologists contains articles on many of the points above.

Software and code

Policy information about availability of computer code

Data collection	RT-qPCR data collection was performed using CFX Maestro 1.1 version 4.1.2433.1219.
Data analysis	All statistical analysis was performed in Graphpad Prism v8.2.1., with the exception of the mAb quantification in Figure 4 which was performed using Graphpad Prism v7.0.4. RT-qPCR analysis was performed using CFX Maestro 1.1 version 4.1.2433.1219.

For manuscripts utilizing custom algorithms or software that are central to the research but not yet described in published literature, software must be made available to editors/reviewers. We strongly encourage code deposition in a community repository (e.g. GitHub). See the Nature Research guidelines for submitting code & software for further information.

Data

Policy information about availability of data

All manuscripts must include a data availability statement. This statement should provide the following information, where applicable:

- Accession codes, unique identifiers, or web links for publicly available datasets
- A list of figures that have associated raw data
- A description of any restrictions on data availability

The datasets used and/or analyzed during the current study are available from the corresponding author on reasonable request.

Field-specific reporting

Please select the one below that is the best fit for your research. If you are not sure, read the appropriate sections before making your selection.

☒ Life sciences ☐ Behavioural & social sciences ☐ Ecological, evolutionary & environmental sciences

Life sciences study design

All studies must disclose on these points even when the disclosure is negative.

Sample size	For Ebola-Zaire "Kikwit isolate", assuming a one-tailed alpha of 0.05, sample sizes of 5 per group will provide >80% power to detect a difference in proportion of surviving animals between the treatment group (100% survival rate) and the control group (0% survival rate), using a Fisher's exact test. One control animal per challenge period is used to confirm lethality of challenge material used in the treatment studies where-in clinical parameters are compared with historical control animals derived from other experiments using the identical challenge material (ie same virus passage). This allows for an ethical reduction in animal number use for data with highly predictable, lethal outcomes.
Data exclusions	No data was excluded from the analysis.
Replication	Due to the ethical and technical challenges experiments involving animals, and specifically non-human primates present in BSL-4 conditions, it was not feasible to conduct multiple experimental repetitions for this study.
Randomization	Animals were issued a number from 1-16, and randomly assigned to groups using a random number generator.
Blinding	Due to the academic nature of this study, as well as the inherent challenges in designing and staffing blinded studies in ABSL-4 settings, blinding was not performed for this study, as it was not necessary to answer the questions set forth by the study.

Reporting for specific materials, systems and methods

We require information from authors about some types of materials, experimental systems and methods used in many studies. Here, indicate whether each material, system or method listed is relevant to your study. If you are not sure if a list item applies to your research, read the appropriate section before selecting a response.

Materials & experimental systems

n/a	Involved in the study	
☐	☒	Antibodies
☐	☒	Eukaryotic cell lines
☒	☐	Palaeontology
☐	☒	Animals and other organisms
☒	☐	Human research participants
☒	☐	Clinical data

Methods

n/a	Involved in the study	
☒	☐	ChIP-seq
☒	☐	Flow cytometry
☒	☐	MRI-based neuroimaging

Antibodies

Antibodies used

Rabbit anti-EBOV VP40 pAb (IBT Bioservices, Cat # 0301-010, Lot # 1107001)
Goat anti-rabbit IgG biotinylated pAb (Vector labs, Cat# BA-1000, Lot # ZE1218)
Goat anti-Human IgG FC-HRP pAb (Invitrogen, Cat# A18817, Lot# 58-101-022118)
Goat anti-Monkey IgG HRP pAb (Fitzgerald, Cat# 43R-IGO20-HRP, Lot# c15032720)
Goat anti-Monkey IgM HRP pAb,(KPL [Seracare], Cat# 5220-0334, Lot# 10371912
Mouse anti-Human IgG (CH2 domain) mAb (Invitrogen, Clone R10Z8E9, Cat# MA5 16929, Lot# U12838159)
MIL77 anti-EBOV antibody cocktail (manufactured by mAbworks for Mapp Biopharmaceutical, experimental antibody treatment for Ebola virus infection, not commercially available)

Validation

Rabbit anti-EBOV VP40 pAb- Validated by manufacturer using western blot and ELISA. Antibody has been validated for use for immunohistochemistry in previous publications from our lab (Mire et al 2015 [PMID: 25853476], Cross et al 2016 [PMID: 27354371], Mire et al 2016 [PMID: 27284090]).

Goat anti-rabbit IgG biotinylated pAb- This antibody has been validated for use by immunohistochemistry by the manufacturer, and has been used in previous publications from our lab (Mire et al 2015 [PMID: 25853476], Cross et al 2016 [PMID: 27354371], Mire et al 2016 [PMID: 27284090]).

Goat anti-Human IgG FC-HRP pAb- The sensitivity of each lot of antibody is confirmed using ELISA by the manufacturer. The specificity of each lot of antibody is confirmed by isoelectric focusing (IEF) by the manufacturer.

Goat anti-Monkey IgG HRP pAb- The antibody has been validated by Western blot by the manufacturer. Data within this manuscript validates its use for ELISA.

Goat anti-Monkey IgM HRP pAb- Each lot is tested by the manufacturer to assure specificity and lot-to-lot consistency using an in-house ELISA assay.

Mouse anti-Human IgG (CH2 domain) mAbMouse anti-Human IgG (CH2 domain) mAb- Validated for use by ELISA by manufacturer, and has been used for ELISA in Gilchuk et al 2018 (PMID: 29982718)

MILL77 is a cocktail of three proprietary humanized mouse monoclonal antibodies directed against Ebolavirus antigens. The cocktail was manufactured by mAbworks for MAPP. Each MIL77 antibody is formulated, individually, in aqueous solution at 25mg/ml protein concentration; 240mM Trehalose dihydrate ; 0.04% Polysorbate 20; 20mM L-Histidine and L-Histidine hydrochloride monohydrate; pH 5.7~6.3. The cocktail is equi-part (1:1:1) for each antibody. Relevant publications: Cao et al 2018 (PMID: 30514891), Qiu et al 2016 (PMID: 26962157)

Eukaryotic cell lines

Policy information about cell lines

Cell line source(s)	Vero E6 (ATCC CRL-1586) was obtained from American Type Culture Collection (ATCC).
Authentication	Independent validation of the Vero E6 cell line was not performed outside of any authentication performed by ATCC.
Mycoplasma contamination	Cells were tested for mycoplasma contamination. No detectable mycoplasma or endotoxin levels were measured (< 0.5 endotoxin units (EU)/ml).
Commonly misidentified lines (See ICLAC register)	No commonly misidentified cell lines were used in the study.

Animals and other organisms

Policy information about studies involving animals; ARRIVE guidelines recommended for reporting animal research

Laboratory animals	Research-naive rhesus macaques (Macaca mulatta, ~3-4 years, 3-6 kgs, balanced sex distribution)
Wild animals	No wild animals were used in the study.
Field-collected samples	No field-collected samples were used in the study.
Ethics oversight	The animal studies were performed at the Galveston National Laboratory, University of Texas Medical Branch at Galveston (UTMB) and were approved by the UTMB Institutional Animal Care and Use Committee. This facility is fully accredited by the Association for Assessment and Accreditation of Laboratory Animal Care International.

Note that full information on the approval of the study protocol must also be provided in the manuscript.
